# Lens Identification to Prevent Radiation-Induced Cataracts Using Convolutional Neural Networks

**DOI:** 10.1007/s10278-019-00242-y

**Published:** 2019-06-20

**Authors:** Ross Filice

**Affiliations:** MedStar Georgetown University Hospital, 3800 Reservoir Road NW, CG201, Washington, DC 20007 USA

**Keywords:** Deep learning, Convolutional neural network, Object detection, Cataracts, Quality, Population health

## Abstract

Exposure of the lenses to direct ionizing radiation during computed tomography (CT) examinations predisposes patients to cataract formation and should be avoided when possible. Avoiding such exposure requires positioning and other maneuvers by technologists that can be challenging. Continuous feedback has been shown to sustain quality improvement and can remind and encourage technologists to comply with these methods. Previously, for use cases such as this, cumbersome manual techniques were required for such feedback. Modern deep learning methods utilizing convolutional neural networks (CNNs) can be used to develop models that can detect lenses in CT examinations. These models can then be used to facilitate automatic and continuous feedback to sustain technologist performance for this task, thus contributing to higher quality patient care. This continuous evaluation for quality purposes also surfaces other operational or process-based challenges that can be addressed. Given high-performance characteristics, these models could also be used for other tasks such as population health research.

## Introduction

Exposure of the lenses to radiation during computed tomography (CT) examinations is of particular concern because it predisposes patients to cataract formation [1,2] and recent estimates of threshold dose appear to be lower than previously thought [3]. Besides avoiding unnecessary CT or other ionizing radiation examinations, there are several methods to avoid or at least decrease lens radiation exposure.

One method utilizes shields that are placed over the eyes to block the direct CT X-ray beam [4, 5], but this is a relatively recent development and is not widely used, particularly in the USA. The other methods use positioning to exclude the lenses; in the case of head CT, this is accomplished by angling the slice acquisitions parallel to a line drawn between the supraorbital ridge and the posterior margin of the foramen magnum [6] in order to include brain parenchyma while excluding the lenses. One way to accomplish this is to tilt the CT gantry and the other is to position the patient by tilting the head forward and tucking the chin to the chest [7]. The focus of this study is head CT examinations, but similar modified principles could be applied to other examinations such as neck CT. In some cases, such as facial bone or sinus CT, avoiding direct lens exposure is impossible using gantry tilt or positioning techniques and must be weighed against the diagnostic benefit of the exam or performed with lens shielding.

Gantry tilt and patient positioning are part of basic CT technologist training, but it has long been observed that compliance is generally low [8], and we have also observed fairly consistent lack of compliance at our institution. This is likely because the techniques required to exclude the lenses from the direct CT beam can be challenging, especially when patient positioning is the only option.

It has also been shown that continuous feedback generally improves and sustains performance [9], and without such feedback, human nature is to accomplish the goal in the most straightforward manner possible. Indeed, we performed a manual quality improvement project at our institution several years prior to this work to optimize lens exclusion in head CT examinations for accreditation purposes; during the project, performance improved substantially but because feedback was manual and cumbersome, it was not sustained and performance returned to baseline when the project concluded. We also found that without such continuous evaluation, department leadership may not be aware of important operational decisions and purchases that could help technologists improve their performance.

**Fig. 1:**
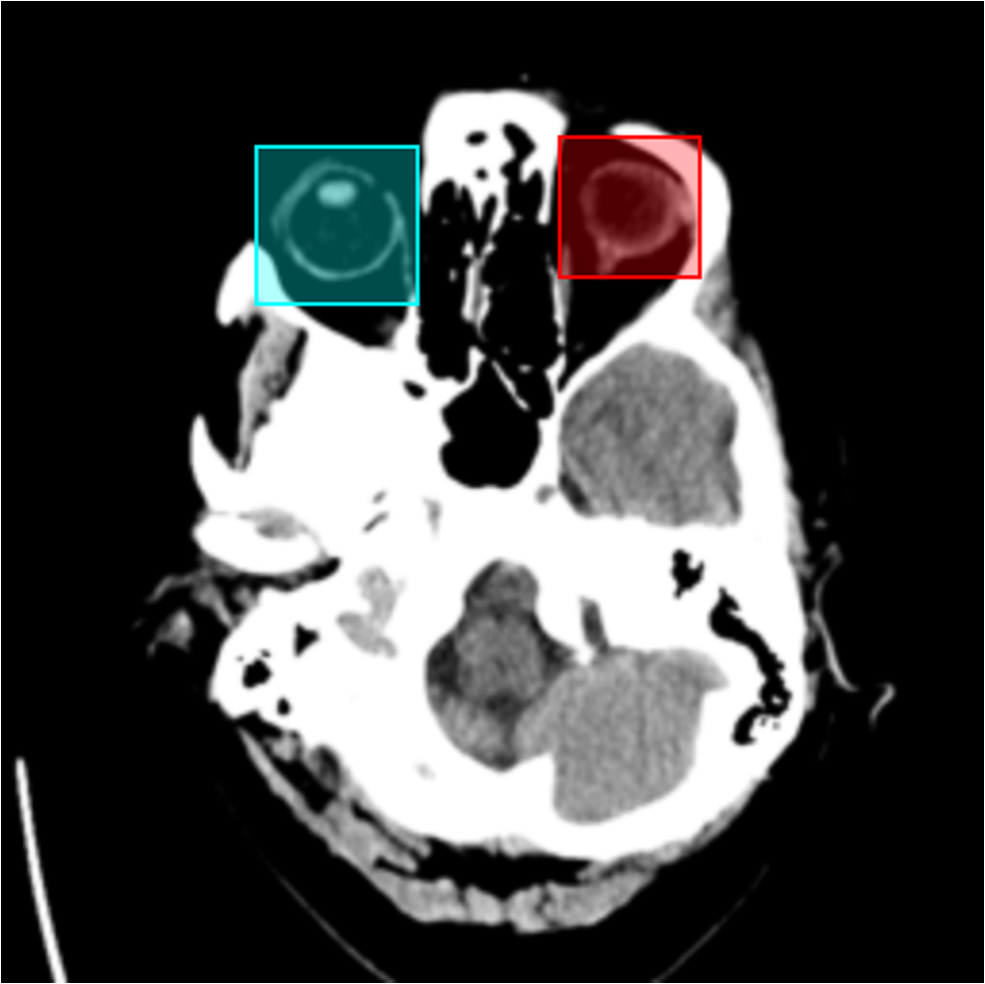
Simultaneous object detection of lens and globe in a representative axial head computed tomography (CT) examination slice

Recently, we developed a deep learning (DL) object detection algorithm utilizing a convolutional neural network (CNN) that can detect both globes and lenses simultaneously and automatically in head CT examinations (Fig. 1). We chose to target head CT examinations because we found that lenses are frequently included but often can be excluded, therefore providing opportunity for substantial quality improvement. Other examinations such as maxillofacial CTs cannot be performed without including the lenses, and examinations such as neck or cervical spine CTs may rarely include the lenses but not enough where intervention would substantially improve performance. We hypothesize that such a model can reliably detect when lenses are included in head CT examinations to both establish objective baseline compliance rates as well as provide prospective real-time continuous feedback to improve compliance. We also believe that this tool could also be used for other purposes such as retrospective population health evaluation.

## Materials and Methods

Institutional review board (IRB) exemption was obtained. One hundred routine head CT examinations consisting of 4128 distinct 512 × 512 3 mm axial soft tissue kernel images were used for training purposes. Thin slice (1–1.5 mm reconstructions), multiplanar reconstructions (coronal and sagittal), and other kernels (bone, etc.) were not used as lenses are readily observed on standard axial images. Pixel data was extracted from the source Digital Imaging and Communications in Medicine (DICOM) files into Portable Network Graphics (PNG) format. Rectangular training bounding boxes were drawn on the objects of interest using a modified version of the open source Simple Image Annotator [10, 11]. Objects of interest included lens, globe, lens implants, and other (i.e., objects near the globes that should be ignored). Labels and bounding box coordinates were stored using the Karlsruhe Institute of Technology and Toyota Technological Institute (KITTI) [12] format.

The pretrained Berkeley Vision and Learning Center (BVLC) GoogLeNet model [13] along with a network modified from the original design of single object detection to allow simultaneous detection of both globes and lenses while ignoring artificial lens implants and other structures was used. Training was performed using the Nvidia Deep Learning GPU Training System (DIGITS) platform [14; version 6.1.0] utilizing the Nvidia fork [15; version 0.15.14] of the BVLC Caffe framework [16, 17]. The Adaptive Moment Estimation (ADAM) solver was used with a base learning rate of 0.0001. No data augmentation or other image preprocessing was performed. Training was performed on a standard workstation equipped with an Nvidia Quadro P6000 graphics processing unit (GPU), but processing of individual images for testing or in real-time quality improvement operations only required a standard central processing unit (CPU) without any specialized processing or deep learning capabilities.

Training was optimized on the 100 examination, 4128 image dataset described above with 826 images (20%) used for in-training validation. Mean average precision (mAP) was used as the performance metric against the validation set. Improvement in mAP was seen fairly quickly after 20–30 epochs with stabilization out to 100 epochs. A separate naïve test dataset of 10 head CT examinations consisting of 404 distinct images was then used to establish accuracy, sensitivity, and specificity for both globes and lenses.

Confidence was established in our model based on the mAP improvement and stability during training as well as our preliminary test results. We then deployed our algorithm operationally for quality improvement purposes. Because of the challenges associated with proper gantry tilt or patient positioning, we chose to focus primarily on outpatient head CT examinations, but with relatively low volume of these types of studies at our institution, we also monitored emergency room (ER) patients. Taking this into account, we first set out to establish baseline performance of our technologists. One hundred thirty-five of our most recent outpatient head CT examinations were evaluated with our algorithm, and compliance was measured on a per-exam and per-technologist basis.

Our intervention consisted of deploying the same model prospectively. Each night we queried our operational report database to find outpatient and ER head CT examinations using radiology information system (RIS) procedure codes. For each exam found, we queried our DICOM archive, moved each exam to our operational server, extracted the pixel data for only the 3 mm axial soft tissue series, evaluated each image for presence of lenses, and logged all pertinent exam and technologist information in a MySQL (Oracle) database. Weekly reports were sent out to our lead CT technologist, physician modality lead, operational modality lead, and department operational manager with compliance metrics as well as examples of good patient positioning on scout images that resulted in exclusion of lenses. Reports were framed as constructive group feedback without isolating individual technologists, though after a period of time we did send individual technologist performance confidentially to our lead technologist so he could work directly with those that continued to underperform.

Finally, after several months of operational deployment, we randomly chose 100 examinations of the 2406 that had been processed through our model, 50 each that were labeled as including or excluding the lenses. We then performed a blinded evaluation of accuracy, sensitivity, and specificity on an exam level for these 100 examinations.

## Results

### Object Detection Model

On an image-level and for the most relevant object detection task, identification of lenses, we found 45 true positives (TP), 1 false positive (FP), 356 true negatives (TN), and 2 false negatives (FN) for sensitivity of 95.7%, specificity of 99.7%, and accuracy of 99.3% (Table 1). For the detection of globes, which could be considered a reasonable proxy for lenses or near inclusion of lenses, we found 66 TP, 1 FP, 335 TN, and 2 FN for sensitivity of 97.0%, specificity of 99.7%, and accuracy of 99.3% (Table 2). One of our lens false positives was an intraocular lens implant in a patient who had cataract lenses surgically removed; this could be considered reasonable feedback as optimal positioning still should exclude this portion of the globe. One of our globe false positives was a lens which could be considered a partial true positive since this is still part of the globe complex. Additionally, our two false positives (one each for eye and lens) had relatively lower confidence scores and thus could be excluded by threshold if specificity was the primary goal for this detection task. For all 10 exams in the dedicated test dataset, we detected the lenses in multiple images for exam-level accuracy of 100%.

**Table 1:** Image-level performance characteristics for the detection of lenses

		Model	
		Lens	None
Truth	Lens	45	2
	None	1	356

**Table 2:** Image-level performance characteristics for the detection of globes

		Model	
		Globe	None
Truth	Globe	66	2
	None	1	335

Testing on 100 random examinations, 50 each labeled as including or excluding lenses, showed exam-level sensitivity of 97.8%, specificity 92.5%, and accuracy 95%. If intraocular lens implants are considered reasonable positive findings (as arguably positioning should exclude this anatomy regardless of implant status), sensitivity is 98%, specificity 100%, and accuracy 99% (Table 3). The single exam that resulted in a false negative was an emergency room patient with their head rotated a full 90°; a relative outlier case that our model was not as familiar with.

**Table 3:** Exam-level performance after operational deployment

		Lens	None
		Model	
Truth	Lens	46	1
	None	4	49
		Model (implant adjusted)	
Truth	Lens	50	1
	None	0	50

The first table is for detection of native lenses; the second for detection of either native or intraocular lens implants.

### Baseline Technologist Compliance

**Fig. 2:**
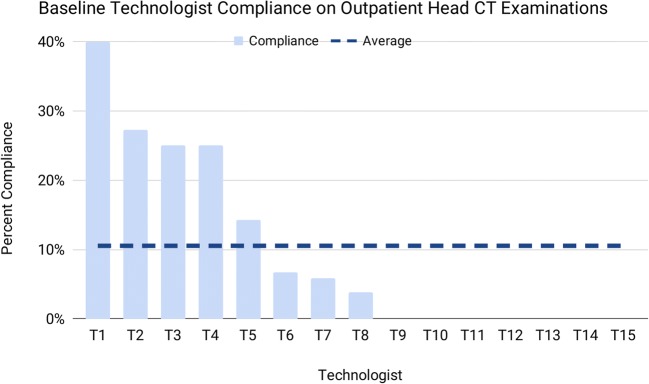
Baseline technologist compliance on outpatient head computed tomography (CT) examinations where compliance is defined as lenses excluded from all axial CT images

**Fig. 3:**
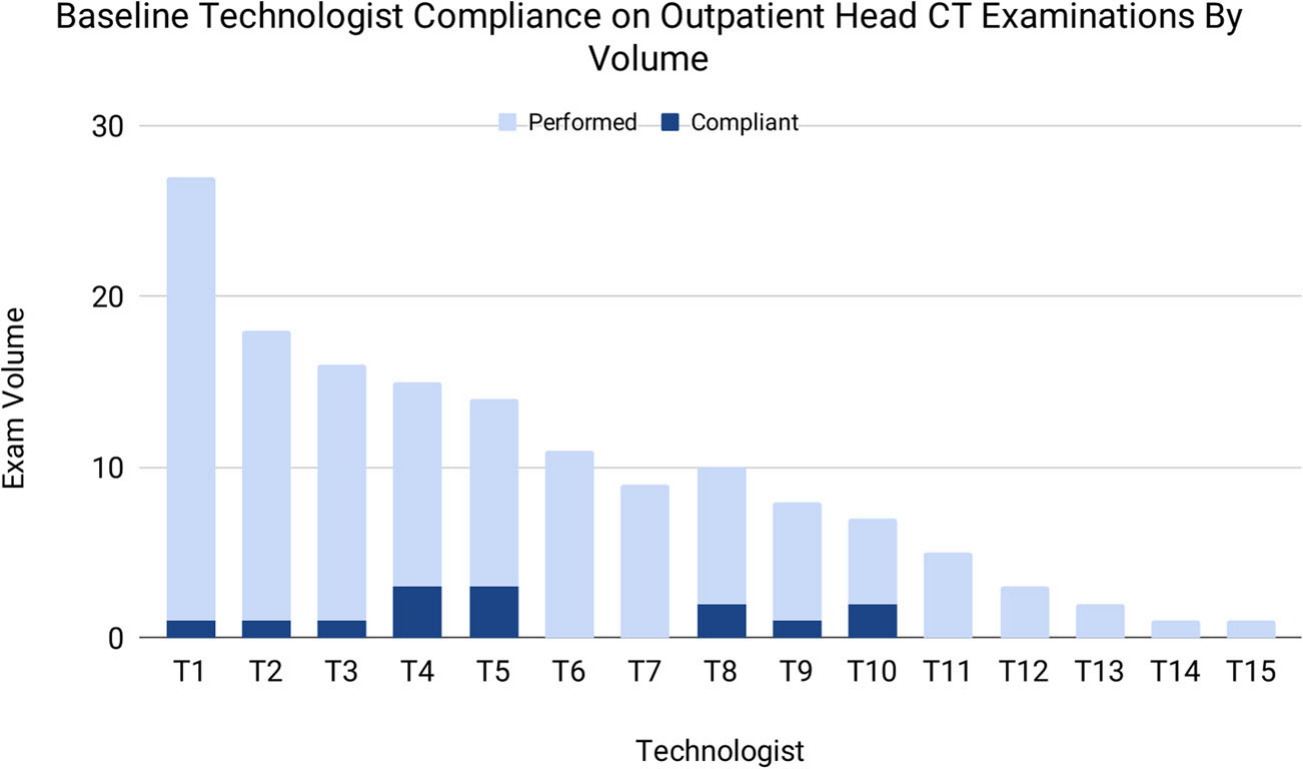
Baseline technologist compliance and examination volume for outpatient head computed tomography (CT) examinations

Baseline compliance on our retrospective review of 135 examinations revealed an average compliance (defined as exams without lenses included in any of the axial 3 mm soft tissue images) of 10.53% with a range of 0–40% (Fig. 2). When evaluating actual numbers of exams performed in context with those exams that were compliant, it became clear that none of the technologists stood out as outliers, good or bad, but that lack of compliance was fairly consistent across the group (Fig. 3). This fits with our general hypothesis that without continuous feedback on this relatively challenging problem, compliance would be low.

### Technologist Performance After Intervention

**Fig. 4:**
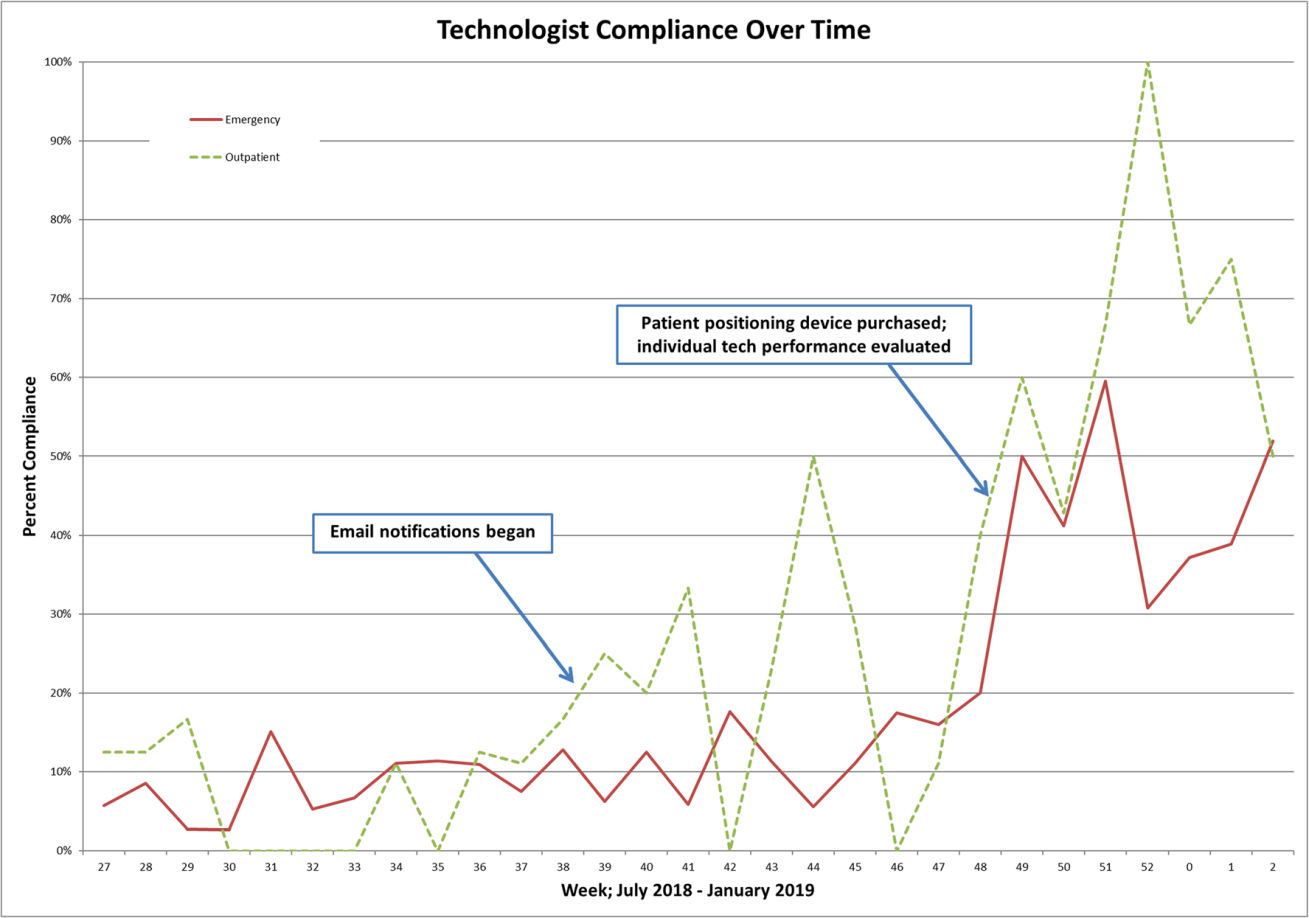
Technologist compliance over time with identification of major intervention time points

We evaluated technologist compliance on a weekly basis with a baseline period of 11 weeks prior to the beginning of email notifications. As above, our focus was on outpatient head CT examinations but we monitored ER head CT examinations as well. Similar to our preliminary results described above, we observed baseline rates of compliance around 10% for both patient classes (Fig. 4).

**Fig. 5:**
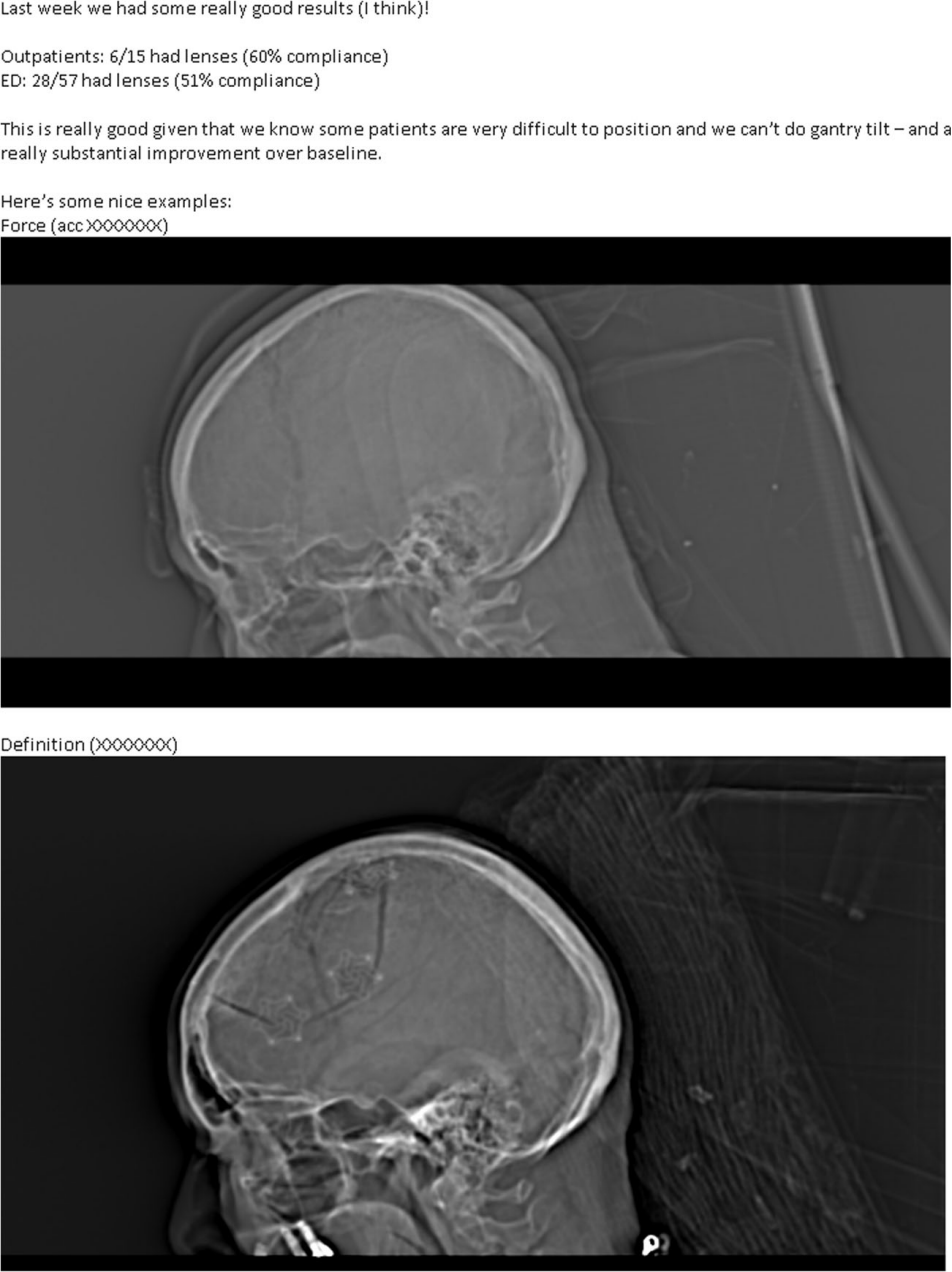
Representative weekly feedback e-mail with statistics, constructive criticism, and representative scout images showing optimal positioning for both computed tomography (CT) scanners

Our email notifications started in week 38 (September) of 2018. A representative e-mail would include group statistics, positive feedback where applicable, and example scout images from examinations on different scanners where positioning worked and the lenses were appropriately excluded (Fig. 5).

Shortly after beginning these e-mail notifications, we held a meeting to discuss the project and any limitations with our lead CT technologist, physician modality lead, operational modality lead, department operational manager, and our chief of neuroradiology. We realized that there were several operational and technical barriers to achieving our quality goal that came to light because of this project. One was that our new dual-energy CT scanner, obtained after our previous manual quality improvement project, did not have gantry tilt capability. We had also stopped using gantry tilt on our other scanner for consistency across both scanners but also because this prevented automatic coronal and sagittal reconstructions due to software limitations. This meant only patient positioning was available to avoid lens inclusion on our scanners which is more difficult to perform properly without gantry tilt.

Our new dual-energy scanner had a built-in patient positioning device which allowed fairly consistent head tilt but our older scanner did not. We decided that we would not start using gantry tilt on our old scanner because of the need to have rapid and automatic reconstructions, but we chose to purchase a patient positioning device for that scanner to mimic the newer patient positioning device and to give our technologists the best chance to achieve our goal. Our new patient positioning device arrived in week 48 (early December) of 2018 which coincided with when we evaluated individual technologist performance with our lead technologist so he could review exams with individuals, especially underperformers.

We observed slow improvement in compliance after our e-mail notifications began with further and substantive improvement after our patient positioning device arrived and after we reviewed individual technologist performance. Our most recent compliance levels for both outpatient and ER patients are averaging between 50 and 60% with higher rates seen in the outpatient class (Fig. 3).

## Discussion

We have developed a reliable simultaneous object detection DL model for both globes and lenses in CT examinations. Because of the nature of human performance, this can be used as an important real-time and continuous quality control feedback mechanism to encourage and remind technologists to use appropriate techniques to avoid lens exposure to direct radiation as much as possible.

An interesting result of this quality improvement project, as in many similar real-world implementations, was the operational limitations and issues we discovered. Our institution has one scanner that does not allow gantry tilt, and gantry tilt was not being used on our other scanner for consistency and software reasons. This limited our technologists’ capacity to position patients to avoid lens exposure more than we anticipated. However, this also prompted us to purchase a relatively inexpensive patient positioner for our older CT scanner which likely helped improve compliance; we never would have realized this without engaging in this quality improvement project.

Regardless, it appears that having access to automatically generated and continual feedback regarding lens inclusion in head CT examinations helped us substantially improve performance. It is difficult to completely distinguish the effects of the automated group e-mails from individual information provided to our lead technologist; similarly operational decisions such as our new patient positioner and simple heightened awareness likely contributed. However, we are confident that this new DL technology allowed us to both improve introspection and facilitate better technologist performance.

One interesting additional issue that arose at the end of this project was that our neurosurgery section complained about head CT examinations performed on patients with substantial head tilt. They felt that the variable angle of head tilt and resultant variable orthogonal reconstructions made it difficult for them to plan procedures and operations. We are currently in the process of evaluating whether we can perform head tilt and reconstructions in a consistent manner, or adjust our reconstruction techniques, such that they will be satisfied with the imaging, or whether this might preclude us from performing head tilt maneuvers at least in neurosurgery patients.

While not explored in this study, we believe our model performance is good enough such that population health studies could be performed retrospectively to identify patients at risk for cataract development. Specificity can be even further improved in this use case as appropriate by incorporating confidence scores generated by our object detection model.

Limitations to this study include that it was performed at a single institution, it was difficult if not impossible to control all variables that may have contributed to technologist performance, and evaluation for lens inclusion was performed after the scan. We also found that our model was very accurate for detecting native lenses but was somewhat overly sensitive to intraocular lens implants. This is arguably acceptable both because head positioning and gantry tilt still should exclude this anatomy, and also because a quality control project generally favors sensitivity over specificity, but this still could likely be improved upon. We also found one false negative exam where the patient’s head was rotated a full 90°; this is an unusual case but something that could be trained for either with additional data collection or data augmentation (specifically rotation) prior to training.

Further data collection, particularly patients with intraocular lens implants and from other sites or outside institutions, might better generalize our model and improve performance. There may be unforeseen benefit in including thin slice or reconstructed datasets in addition to standard 3 mm axial images which we did not explore. For the quality improvement portion, one might perform a modified version of this study at another site or institution while attempting to control for each interventional variable (group notifications, individual performance reviews, operational decisions and purchases, etc.), but this would be more challenging and we are not particularly confident this would result in substantial improvement beyond what we have already explored in this project.

Integration of a lens detection predictive model at the time of patient positioning or scout image acquisition could prevent lenses from being included in the first place. This would require a different type of model, whether DL or other, and would also require integration with proprietary scanner software and hardware.

## Conclusions

Deep learning object detection models have real-world utility to improve quality of CT scanning and patient care. Similar models may be integrated with CT scanner software and hardware to prevent lenses from being included in the scan beforehand. There may also be a role for this or similar DL models in population health studies.
